# Reforestation in southern China: revisiting soil N mineralization and nitrification after 8 years restoration

**DOI:** 10.1038/srep19770

**Published:** 2016-01-22

**Authors:** Qifeng Mo, Zhi’an Li, Weixing Zhu, Bi Zou, Yingwen Li, Shiqin Yu, Yongzhen Ding, Yao Chen, Xiaobo Li, Faming Wang

**Affiliations:** 1 Key Laboratory of Vegetation Restoration and Management of Degraded Ecosystems, South China Botanical Garden, Chinese Academy of Sciences, Guangzhou 510650, P.R. China; 2 University of Chinese Academy of Sciences, Beijing 100049, P.R. China; 3 Xiaoliang Research Station for Tropical Coastal Ecosystems, Chinese Academy of Sciences, Maoming 525029, P.R. China; 4 Department of Biological Sciences, State University of New York-Binghamton, Binghamton, NY 13902, USA; 5 Agro-Environmental Protection Institute, Ministry of Agriculture, 300191 Tianjin, P.R. China

## Abstract

Nitrogen availability and tree species selection play important roles in reforestation. However, long-term field studies on the effects and mechanisms of tree species composition on N transformation are very limited. Eight years after tree seedlings were planted in a field experiment, we revisited the site and tested how tree species composition affects the dynamics of N mineralization and nitrification. Both tree species composition and season significantly influenced the soil dissolved organic carbon (DOC) and nitrogen (DON). N-fixing *Acacia crassicarpa* monoculture had the highest DON, and 10-mixed species plantation had the highest DOC. The lowest DOC and DON concentrations were both observed in *Eucalyptus urophylla* monoculture. The tree species composition also significantly affected net N mineralization rates. The highest rate of net N mineralization was found in *A. crassicarpa* monoculture, which was over twice than that in *Castanopsis hystrix* monoculture. The annual net N mineralization rates of 10-mixed and 30-mixed plantations were similar as that of N-fixing monoculture. Since mixed plantations have good performance in increasing soil DOC, DON, N mineralization and plant biodiversity, we recommend that mixed species plantations should be used as a sustainable approach for the restoration of degraded land in southern China.

The conversion of land from natural forest ecosystems to agricultural ecosystems is the major cause of the current global biodiversity loss^1^. As a main type of land use change, deforestation has led to millions of hectares of degraded or abandoned lands in the last several decades^2^,^3^,^4^, which resulted in global warming by releasing significant amounts of CO_2_ to the atmosphere^2^,^5^,^6^. Hence, reforestations in degraded lands are proposed to increase carbon sequestration, mitigate climate change and restore native ecosystems^7^,^8^

Nitrogen (N) availability plays a central role for tree growth in afforestation practices^8^,^9^. N mineralization is an important microbial mediated process^10^,^11^. Previous documents stated that the rate of N mineralization was primarily controlled by the microbial community composition and activity^12^,^13^. The activities of microbe are, however, predominantly determined by the amount of litter input and root exudate^14^,^15^, in addition to soil pH, soil water contents, temperature and others^16^,^17^,^18^. Since the tree species may result in various physicochemical properties of litter input and root exudate^11^,^19^,^20^,^21^, the tree species compositions are essential for microbial mediated N mineralization in the regenerating forests.

Previous studies showed that tree species composition was a major factor affecting N turnover in various vegetation types globally^15^,^19^,^22^. Generally, N-fixing tree species are able to increase the input of N contents in soil and litter fall, thus affect the soil microbial community and N mineralization underneath these trees^8^. In a 14-year old plantations, Hoodmoed, *et al.*^8^ reported that the total N of leaves and litter in two N-fixing tree species (*Acacia dealbata* and *A. implexa*) were significantly higher than that in two non-N-fixing species (*Eucalyptus camaldulensis* and *E. polyanthemos*). In a 23-year old plantations, Wang, *et al.*^23^ reported that N-fixing *Acacia auriculiformis* and *Acacia mangium* produced relatively higher mineral N than *Eucalyptus urophylla* and *Castanopsis hystrix*^24^. Hence, the plantations dominated by N-fixing tree species might be better than non-N-fixing species in rising soil N availability since N-fixing trees could increase the N contents of leaf, litter and soil^8^,^9^.

In the early stage of reforestation, fast-growing species like *Eucalyptus* and *Acacia* are often recommended for restoring degraded soil and for timber production^23^,^25^,^26^,^27^. Although monoculture plantation has been commonly used for rebuilding the forest ecosystems and increasing the amount of wood products, their influences on soil nutrient availability and ecosystem sustainability are often debatable^8^,^23^. Some studies argued that fast-growing monocultures were generally disadvantageous for increasing soil nutrient availability and enhancing ecosystem function of species diversity^25^,^27^,^28^, and that mixed plantations should be recommended in reforestation instead^25^. For example, Wang, *et al.*^23^ reported that the net N mineralization of 10-mixed species plantation was over two folds higher than that of *Eucalyptus urophylla* monoculture in a two-year restoration plantation. It is believed that mixed species plantations had the potential to increase productivity while maintaining soil fertility compared with monocultures because of the complementarity of resource and nutrient use strategies from the different tree species within the mixed plantations^9^.

Stand age was also an important factor affecting the nutrient cycles in the regenerating plantations. Our previous studies indicated that the pros and cons of fast-growing species on soil nutrient availability and ecosystem development should be evaluated with stand ages^23^,^24^. For example, in two-year-old plantations, Wang, *et al.*^23^ found that monoculture *Eucalyptus* and *Acacia* plantations had lower soil N mineralization rates and reduced N leaching loss relative to 10- and 30- species mixed stands. However, in another study, they investigated soil nutrients availability in 23-year-old plantations, and found that *Eucalyptus* and *Acacia* monoculture had higher, or at least equal, soil N transformations rates than the native species plantations^24^.

Because of the strong effects of tree species composition and age on ecosystem development, here, we investigated soil net N mineralization and nitrification rates of six eight-year-old plantations in a controled forest experiment in southern China. We test the following three hypotheses: 1) mixed plantations would have higher soil net N mineralization and nitrification rates than monoculture; 2) Due to additional N input, net N mineralization rate of N-fixing species would be higher than that of non-N-fixing species; 3) soil net N mineralization and nitrification rates of plantations would vary with seasons and stand ages, due to the seasonal variation of temperature and rainfall and the plant growth with litter feedback.

## Results

### Soil general properties

Vegetation characteristics of six plantation types were shown in Table 1. After 8 years of plant growth, the pH, soil organic matter (SOM), total N, total P and available P in the 0-10 cm soil layer were all significantly affected by the experimental plantation types (Table 2). The shrubland (SL: unplanted control) had the highest soil pH among the six treatments, while *Acacia crassicarpa* (AC) had the lowest pH. Both of SL and AC had higher SOM contents than others. The *Ecucalyptus urophylla* (EU) had the lowest soil total N and highest C:N ratios among all treatments (Table 2). Both of 10- and 30-species mixed plantations sustained relatively higher amount of available P than others, while EU had the lowest soil available P.

**Figure 1:**
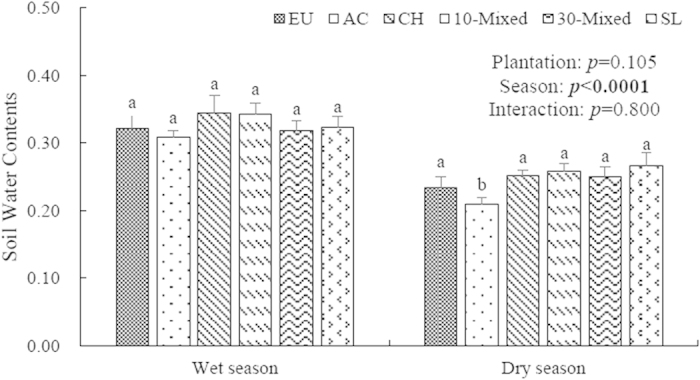
Soil water contents (SWC) in upper 0–10 cm soil layer of six plantations at Heshan Station in 2013. Different lowercase letters denote significant differences in six plantations, n = 9, *p* < 0.05. EU, *Ecucalyptus urophylla* monoculture; AC, *Acacia crassicarpa* monoculture; CH, *Castanopsis hystrix* monoculture; 10-mixed, 10 species mixture; 30-mixed, 30 species mixture; SL, unplanted shrubland.

There was significantly seasonal effects on soil water contents (SWC) among six plantations (*p* < 0.001), but neither plantation nor plantation ×season interactive effects on the SWC was found (*p* = 0.105 and *p* = 0.800, respectively) (Fig. 1).

### Soil inorganic N

**Figure 2:**
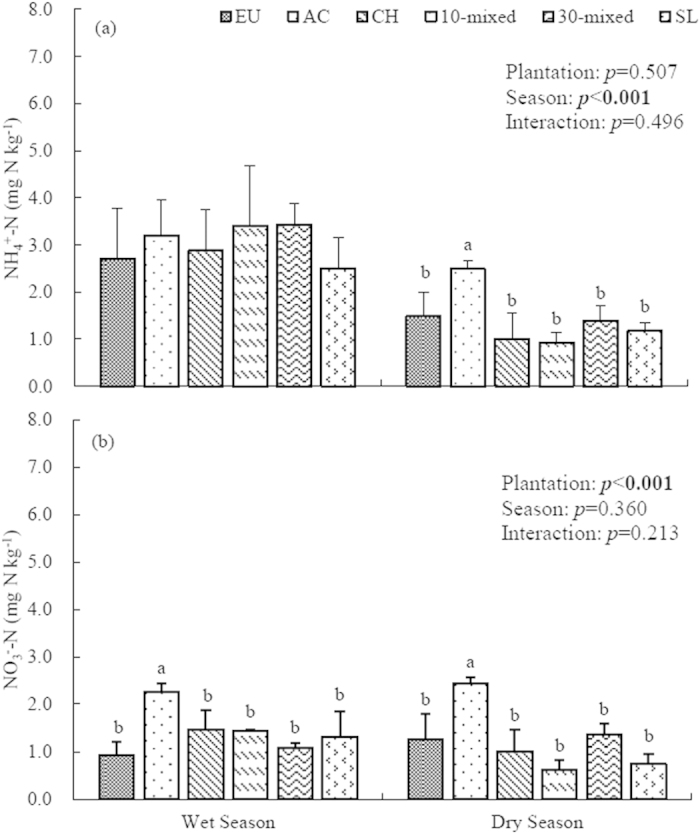
Concentrations of NH_4_+-N (**a**) and NO_3_^−^-N (**b**) in upper 0–10 cm soil layer of six plantations at Heshan Station, 2013. Different lowercase letters denote significant differences in six plantations, n = 9, *p* < 0.05. EU, *Ecucalyptus urophylla* monoculture; AC, *Acacia crassicarpa* monoculture; CH, *Castanopsis hystrix* monoculture; 10-mixed, 10 species mixture; 30-mixed, 30 species mixture; SL, unplanted shrubland.

Plantation type significantly affected the concentrations of NO_3_^−^-N in both the wet and dry season, but affected the concentrations of NH_4_^+^-N in the dry season (Fig. 2). The concentrations of NH_4_^+^-N were generally higher than those of NO_3_^−^-N, indicating that NH_4_^+^-N was the major form of inorganic N in our study site. In the wet growing season, the NH_4_^+^-N concentrations of the AC, 10- and 30-species mixed plantations were similar (3.19, 3.41, 3.44 mg N kg^−1^, respectively), while the SL had the lowest NH_4_^+^-N concentration 2.50 mg N kg^−1^. The NO_3_^−^-N concentration of the AC was significantly higher than other plantations with the value of 2.27 mg N kg^−1^ (Fig. 2). In the dry season, both the NH_4_^+^-N and NO_3_^−^-N concentrations of the AC plantation were significantly higher than those of other treatments, with the values of 2.49 and 2.27 mg N kg^−1^, respectively (Fig. 2).

The concentrations of total inorganic N (the sum of NH_4_^+^-N and NO_3_^−^-N) in the wet season were significantly higher than in the dry season across all six treatments (*p* < 0.0001). The plantation type significantly affected the total inorganic N (*p* < 0.05), and the AC plantation had higher total inorganic N than other five plantations in both the wet and dry season. In addition, the total inorganic N concentrations of 10-species mixed and 30-species mixed were both higher than those of EU, CH and SL treatments (Fig. 2).

### Net N mineralization and nitrification

**Figure 3:**
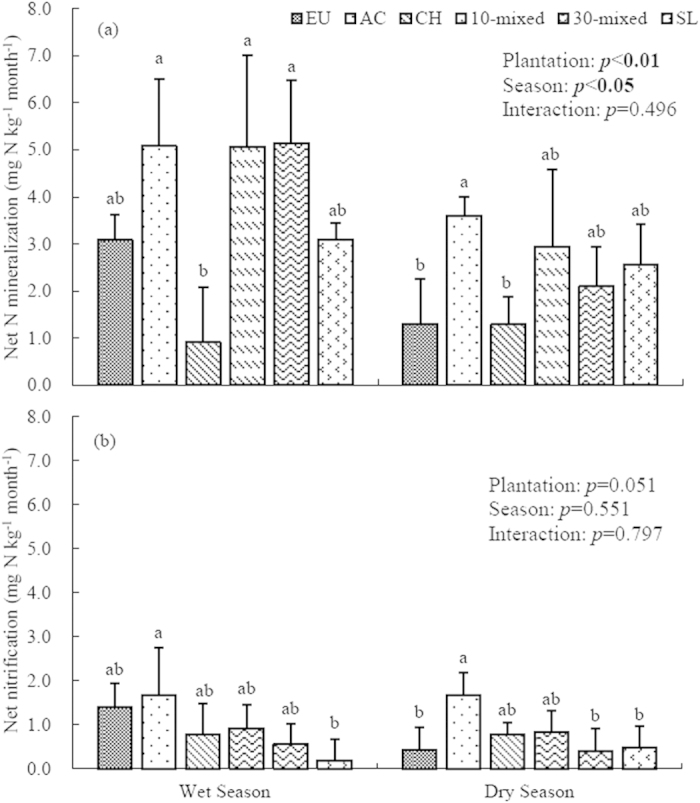
Monthly rates of net N mineralization (**a**) and nitrification (**b**) in upper 0–10 cm soil layer of six plantations at Heshan Station in 2013. Different lowercase letters denote significant differences in six plantations, n = 9, *p* < 0.05. EU, *Ecucalyptus urophylla* monoculture; AC, *Acacia crassicarpa* monoculture; CH, *Castanopsis hystrix* monoculture; 10-mixed, 10 species mixture; 30-mixed, 30 species mixture; SL, unplanted shrubland.

Both plantation type and season had significant effects on the rates of net N mineralization (*p* < 0.05 for both), but no plantation × season interactive effect was found (Fig. 3a, *p* = 0.469). In the wet season, the AC, 10- and 30-species mixed plantations had relatively higher rates of net N mineralization (5.08, 5.07 and 5.14 mg N kg^−1^ month^−1^, respectively), while the *Castanopsis hystrix* (CH) had a lower net N mineralization rate of only 0.91 mg N kg^−1^ month^−1^ (Fig. 3a). EU and unplanted SL stands also had lower net N mineralization rates. In the dry season, net N mineralization rate of AC was 3.60 mg N kg^−1^ month^−1^, which was significantly higher than those of EU and CH monocultures (Fig. 3a).

Plantation types marginally affected the net nitrification rates (*p* = 0.051, Fig. 3b). In the wet season, net nitrification of the AC monoculture was significantly higher than that of unplanted SL. In the dry season, the highest net nitrification rate was also found in the AC (1.67 mg N kg^−1^ month^−1^), which was significantly higher than those of the EU, 30-species mixed, and SL treatments (Fig. 3b).

In this study, to make effective comparison with previous studies, we also estimated annual N mineralization rates by multiplying monthly rates with the respective durations of wet seasons and dry seasons in the region. Plantation type significantly affected annual net N mineralization rates (*p* < 0.01). The annual N mineralization rate of the AC was significantly higher than that of the EU and CH monocultures (Table 3), being 97.5% and 294.6% higher, respectively, revealing that legumes species might maintain greater available N contents than other non-legumes species. Although the annual N mineralization of AC monocultures was 8.1% and 19.9% higher than that of 10-species mixed and 30-species mixed plantations. However, these differences were not statistically significant (Table 4). In addition, the estimated annual N mineralization of AC plantation was 53.5% higher than that of SLtreatment.

The ratios of annual nitrification to N mineralization (N_nit_/N_min_) ranged from 11.91% in SL to 41.75% in EU, with an exception of a relatively higher ratio in CH at 71.42%. Across the six treatments, the ratios of N_nit_/N_min_ of three monocultures were generally higher than those of two mixed plantations and SL (Table 3).

### Soil extractable dissolved organic carbon (DOC) and nitrogen (DON)

**Figure 4:**
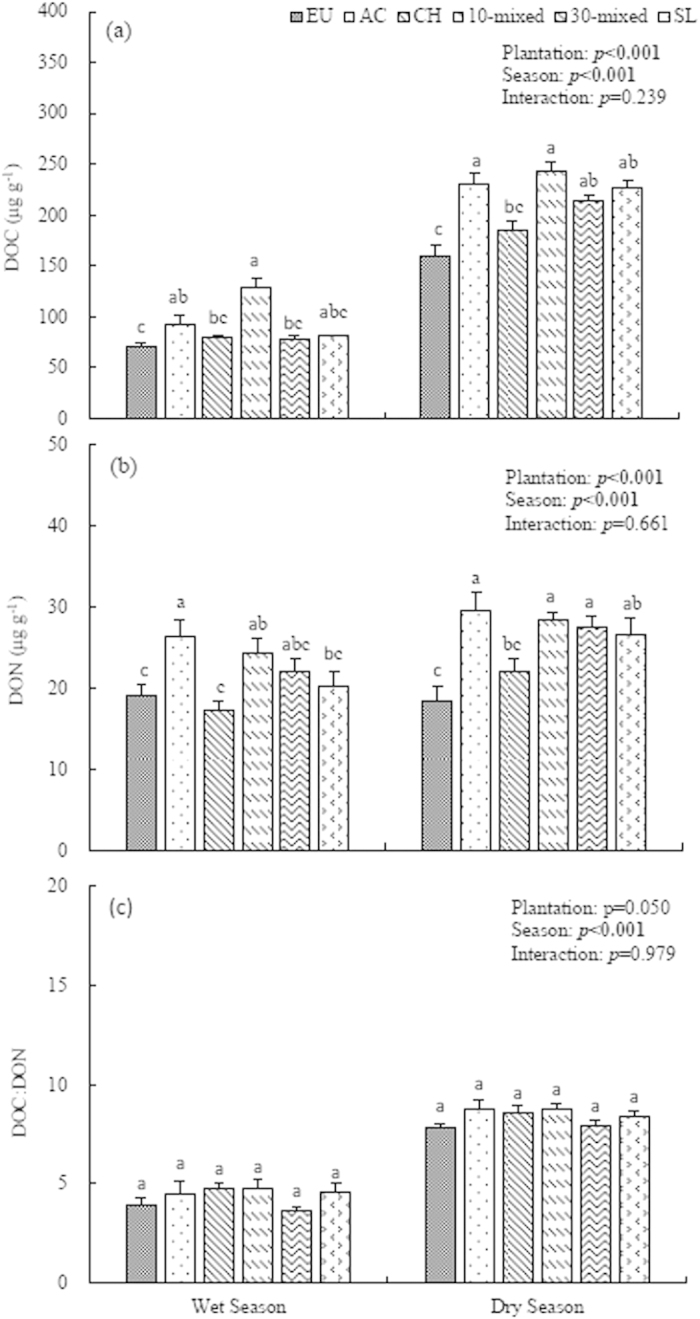
Extractable dissolved organic carbon (DOC), extractable dissolved organic nitrogen (DON) and their ratios (DOC:DON) in soil 0–10 cm layer of the six plantations at Heshan Station, 2013. Different lowercase letters denote significant differences in six plantations, n = 9, *p* < 0.05. EU, *Ecucalyptus urophylla* monoculture; AC, *Acacia crassicarpa* monoculture; CH, *Castanopsis hystrix* monoculture; 10-mixed, 10 species mixture; 30-mixed, 30 species mixture; SL, unplanted shrubland.

The soil extractable dissolved organic carbon (DOC) in the dry season was significantly higher than that in the wet season (*p* < 0.001). The plantation type also significantly affected the DOC concentrations. Our study showed that the 10-species mixed plantation had the highest, while the EU had the lowest DOC concentrations among six plantations in both season. Moreover, AC had the second higher DOC concentration among six plantations (Fig. 4). In addition, both season and plantation type had significant effects on the concentrations of soil dissolved nitrogen (DON) (*p* < 0.001 for both). Not surprisingly, the AC plantation had the highest DON concentration, while the EU and CH monoculture had the relatively lower DON concentrations. Moreover, the mixed plantations maintained relatively higher DON concentrations than EU and CH monocultures (Fig. 4).

Although the DOC/DON ratios in dry season was significantly higher than that in wet season (*p* < 0.001), plantation had a weak effect on DOC/DON ratios (*p* = 0.050). Our results also showed that no plantation×season effect was found on the DOC, DON or DOC/DON ratios (Fig. 4).

## Discussion

Plantation types significantly affected the rates of net N mineralization in our study, which revealed that tree species composition might drive N transformation in plantation ecosystems^15^,^29^. In this study, we found that the net N mineralization rates of N-fixing *Acacia crassicarpa* (AC) plantation were much higher than that of other monocultures. This results agreed with our hypothesis 2 that N-fixing species produced greater potential N than non-N-fixing species. Surprisingly, this result was inconsistent with the previous reports at the same site^23^, which found that AC monoculture had lower N mineralization rate than non-N-fixing native species after two-years planting. However, in another study of 23-year old plantations nearby, Wang, *et al.*^24^ found that the annual net N mineralization of plantations dominated by N-fixing species was relatively higher than those dominated by non-N-fixing species (Table 4). This supported our present results that N-fixing tree species maintained higher net N mineralization than non-N-fixing species. Moreover, in a 13-year-old plantation of the adjacent area, Li, *et al.*^30^ also demonstrated that both the higher nitrogen levels of legume litters and their relatively low rates of decomposition were important factors in the buildup of nitrogen stocks in the soils of legume forests. Strong correlations between N mineralization and soil indices were likely the results of long-term feedbacks between litterfall, microbial mineralization and plant nutrient uptake. Our results, combined with previous studies in the nearby sites (Table 4), suggested that N-fixing species, although did not increase soil N availability in two-year old plantation^23^, could enhanced soil N cycling in a relative longer time frame (8 yrs, 13 yrs and 23 yrs).

The net N mineralization of the *Castanopsis hystrix* (CH, native species) monoculture was the lowest among the six treatments. There were many reasons that might explain the lower N supply in CH plantations: firstly, we observed relatively lower aboveground biomass and vegetation coverage in this plantation (Table 1); secondly, the annual litter input of CH monoculture was *c.* 35% lower than that of AC (Yu *et al.*, unpublished data); thirdly, we found that the CH monoculture had the lowest dissolved organic nitrogen (DON) than other plantations in the study (Fig. 4). Furthermore, the soil respiration of the CH was also relatively lower than that of other treatments in the study (Yu *et al.*, unpublished), suggesting lower microbial biomass and activities in this plantation. In addition, the relatively lower soil pH in AC plantation might have also contributed to the lower microbial activities (Table 2). Since soil microbes greatly regulate the mineral N production by decomposing the organic matter or litterfall^31^, it was thus reasonable that the CH had lower N transformation rates than other treatments.

In this study, the mixed species plantation had the similar net N mineralization rate as the N-fixing AC monoculture. We also observed that relatively higher DON in mixed plantation that would contribute to the microbial activities then to soil N cycling and supply. However, the mixed species plantations were usually more productive, sustainable and essential for the natural forest succession than monocultures because of the complementarity of resource and nutrient use strategy among different tree species within the mixed plantation^9^,^25^,^32^. For example, Montagnini^33^ had suggested that above-ground biomass of mixed plantation was always larger than pure stand in tropical lowland regenerated forests because the less intra-specific competition for resource within the mixed plantation. Although the N-fixing monoculture could acquire additional N for the ecosystem, our results indicated that mixed plantations would have the similar N supply levels as N-fixing monoculture after eight years, which was in line with the hypothesis 1. This may allow mixed species plantations perform better than monocultures in restoring degraded land. Mixed species plantations also benefit greatly to native species diversity and conservation.

Seasonal variation appeared to have a stronger effect on soil N transformations in forest ecosystems^34^,^35^,^36^. In our study, the net N mineralization had a significant seasonal variation: the rates of net N mineralization in the wet season was generally higher than that in the dry season, which was consistent with our hypothesis 3. Seasonal variations of N mineralization and nitrification were often ascribed to seasonal variations of temperature and moisure^23^,^37^, which may affect the decomposition of soil organic matter and nitrogen availability^17^. In our study, soil water contents (SWC) was higher in wet season than in dry season. Thus, the higher soil microbial activities, due to favorable soil water content and higher temperature, would increase N mineralization in wet season^38^.

We also found that the ratios of N_nit_/N_min_ ranged from 11.91–41.75% in five out of six treatments with the exception of a higher ratio in the CH at 71.42% in the eight-year-old plantations. In the same site after two-years initial planting, however, Wang, *et al.*^23^ found that the ratios of annual N_nit_/N_min_ ranged from 73.93–90.00%. The decline of N_nit_/N_min_ ratios following ascending plantation ages is consistent with the hypotheses 3 and the results reported by Maithani, *et al.*^37^, who investigated the comparative N mineralization of 7-,13- and 16-year old forest and found that nitrification rates declined while ammonification rates increased with the stand age in a regenerated forest. Clearly, eight years plant growth generated much higher aboveground biomass, quantity of litter fall, fine root biomass (personal observations) that were very different from the two-year old correspondent^23^. The declining Nnit/Nmin ratios would be associated with the changes in aboveground vegetation and soil properties (i.e., increased soil TN, SOC *et al.*) with ascending stand ages^39^. Further investigation of the underlying mechanisms of tree species composition on soil nutrient cycles will be beneficial to reforestation succession in tropical area of southern China.

## Conclusions

The net N mineralization and nitrification rates in these experimental plantations demonstrated that tree species composition and stand age are important factors influencing soil nutrient availability. As an N-fixing species plantation, *Acacia crassicarpa* monoculture had higher net N mineralization than other monocultures eight-year after the plantations. This result differed from that after the first two years, indicating that N-fixing species, although did not increase soil N availability in their two years old, could improve soil N cycling in a relative longer time frame. *Castanopsis hystrix* monoculture, a native species, had the lowest net N mineralization rate. The mixed species plantations had similar level of soil N mineralization rate as N-fixing AC monoculture. The decline of N_nit_/N_min_ ratios with the ascending stand age would be associated with the changes in vegetation and soil physicochemical properties (e.g., soil organic matter and total nitrogen). Based on these findings, we recommend that mixed plantations with native species and introduced N-fixing species should be a sustainable approach in the forest restoration of southern China.

## Materials and Methods

### Site description

The research was conducted in the Heshan National Field Research Station of Forest Ecosystem (Heshan Station, 112°50′E, 22°34′N), in the subtropical region of southern China. The soil type is Arenosol developed from sandstone, with a pH of about 4.0. The climate of this region is typical subtropical monsoon, with mean annual temperature of 22.6 °C and highest average temperature of 28.7 °C in July and lowest average temperature of 14.5 °C in January. The annual precipitation in this region is 1700 mm and over *c.* 85% rainfall in the wet season. In this region, there is a distinct wet season and dry season. The wet season (from April to September) is hot and wet, while the dry season (from October to March) is cool and dry.

The region is a hilly agricultural zone with 78.6% of hilly land, 17.1% of farming land and 4.3% of water body. The elevation of the studied area is 60.7 m, with gently rolling topography. Although the study plots are randomly distributed in the huge 50 ha area, the soil characteristic and topography are almost identical among all 1-ha plots.

### Experimental design

An ecological restoration project was launched at Heshan Station in 2005. Historical monoculture Masson pine (*Pinus massoniana*) plantation, within an area of 50 ha, was cut down and the residue was burned. Then five types of experimental plantations were established: *Eucalyptus urophylla* (EU) (non-legumes) monoculture; *Acacia crassicarpa* (AC) (legumes) monoculture; *Castanopsis hystrix* (CH) (native) monoculture; 10-species mixture (10-mixed) and 30-species mixture (30-mixed), plus one unplanted control (without any planting and naturally developed into a shrubland, SL). The 10-species mixed plantation included seven native species, *Castanopsis hystrix*, *Liquidambar formosana*, *Machilus chinensis*, *Cinnamomum burmanii*, *Tsoongiodendron odorum*, *Bischofia javanica*, *Schima superba*, and the three species used in monocultures. The 30-species mixed plantation contained 24 native species, *Michelia macclurei*, *Ormosia pinnata*, *Sterculia lanceolata*, *Garcinia oblongifolia*, *Garcinia cowa*, *Dracontomelon dao*, *Elaeocarpus japonicas*, *Cinnamomum parthenoxylon*, *Radermachera sinica*, *Maesa japonica*, *Dolichandrone caudafelina*, *Michelia chapensis*, *Syzygium cumini*, *Elaeocarpus apiculatus*, *Castanopsis fissa*, *Acronychia pedunculata* and *Schefflera octophylla*, and *3 exotic species*, including *Delonix regia*, *Grevillea robusta*, *Pterocareus indicus*. All plants in the 10-species mixture were also used in the 30-species mixture. Each plantation treatment was replicated three times (18 plots in total), randomly distributed in the 50 ha study area. Tree samplings in tube stocks with similar height (50–100 cm) were planted at 2 × 3 m spacing in each plot in 2005. In mixture treatment, different species were placed randomly.

Soil physicochemical properties (0–5 cm layer) were monitored by Wang *et al.*^23^, 2 years after the initial planting across all six plantations, and there was no significant effect of plantation on pH, SOM, total N, total P, available P and C:N ratios, which indicated homogeneity across all the plots.

### Soil sampling and analysis

*In situ* N mineralization incubation was determined in May 2013 (Wet season) and December 2013 (Dry season), using a PVC (polyvinyl chloride plastic) core method (modified from Raison *et al.*, 1987). Specifically, three subplots were randomly located in each replicated plot. In each subplots, 3 sampling point was selected. At each point, two sharpened PVC cores (4.6 cm diameter×15 cm height) were dived 10 cm into the ground, one of the two tubes was retrieved directly and sent to lab (S0) stored at 4 °C immediately, and the other covered with a lid and had some holes on the side wall for aeration (S1), incubated *in situ* for one month (30 days) before retrieved for the same soil analysis. Totally, for each subplot, there was 3 soil cores collected and then mixed thoroughly. For each plantation treatment, there were 9 replicated soil samples (3 plots × 3 subplots).

Soil samples were brought to the laboratory in an ice-box, fresh-sieved at 2-mm mesh removing stones, visible roots and plant residuals and stored at 4 °C for analyses. For inorganic N (NH_4_^+^-N and NO_3_^−^-N) measurement, 10 gram sieved soil before and after the incubation were extracted with 50 ml 2 M KCl. Concentrations of ammonium and nitrate in the filtered extracts (Shuangquan quantitative filter paper 202#) were determined using a flow injection auto analyzer (FIA, Lachat Instruments, Loveland, CO, U.S.A). The subsample was also used for measuring soil extractable dissolved carbon (DOC) and nitrogen (DON). For DOC and DON measurement, 20 grams sieved soil was extracted with 60 ml 0.5 M K_2_SO_4_ solution, and their concentrations were determined using a Shimadzu TOC-V CSH analyzer. Soil water content (SWC) was determined with subsamples being dried at 105 °C for 24 hours.

The air-dried subsamples were used for measuring the pH value, total organic C and N contents. The pH value were determined in the deionized water suspension (water: soil = 2.5:1). Soil total organic C concentrations were measured using H_2_SO_4_-K_2_Cr_2_O_7_ oxidation method^23^. Soil total nitrogen (TN) was determined using the Kjeldahl acid-digestion method with an Alpkem autoanalyzer (Kjektec System 1026 Distilling Unit, Sweden). Soil total P (TP) concentration was measured photometrically after digesting soils with sulfuric acid (H_2_SO_4_).

The rates of net N mineralization were calculated from the differences of inorganic N (NH_4_^+^-N+NO_3_^−^-N) concentrations between the initial and post incubation soil samples. Calculated cumulative net N mineralization and nitrification rates were calculated by summing the rate of net N mineralization of each incubation period during the wet season and dry season.

### Statistical analysis

Two-way ANOVA was performed to test the effects of tree species composition (five plantations and one shrub land), season (wet and dry season) and their interactions on inorganic N (NH_4_^+^-N, NO_3_^−^-N) concentrations, soil dissolved organic carbon (DOC) and nitrogen (DON), net N mineralization and nitrification rates during the experiment period. Least significant differences (LSD) post hoc test was used to compare the effects of planting treatment on the above variables at each season. General soil properties, pH, available P, TN, TP, soil organic matter (SOM), C/N mass ratio were analyzed by One-way ANOVA testing planting treatment. All analyses and computations were performed on SPSS 16.0 (SPSS Inc., Chicago, IL, U.S.A) and Microsoft office software 2013 (Microsoft Crop., Redmond, WA, U.S.A).

## Additional Information

**How to cite this article**: Mo, Q. *et al.* Reforestation in southern China: revisiting soil N mineralization and nitrification after 8 years restoration. *Sci. Rep.* **6**, 19770; doi: 10.1038/srep19770 (2016).

## Figures and Tables

**Table 1:** Vegetation characters of five restoration plantations and one shrubland at Heshan Station (data were collected in 2011, 6 years after initial planting).

Plantations types	Overstory Tree Species					Major Understory Species	
	Name	Height(m)	DBH(cm)	Coverage (%)	Trees/ha	Name	Coverage (%)
*Eucalyptus urophylla*	*E.urophylla*	10.66 ± 1.10	9.27 ± 0.95	65	1463 ± 82	*Rhodomyrtus tomentosa*	25
						*Dicranopteris dichotoma*	40
						*Clerodendrum fortunatum*	8
*Acacia crassicarpa*	*A.crassicarpa*	9.12 ± 0.89	8.73 ± 0.24	88	1896 ± 218	*Rhodomyrtus tomentosa*	26
						*Dicranopteris dichotoma*	33
						*Clerodendrum fortunatum*	6
*Castanopsis hystrix*	*C.hystrix*	5.63 ± 0.08	5.26 ± 0.29	30	1485 ± 459	*Rhodomyrtus tomentosa*	8
						*Dicranopteris dichotoma*	13
						*Clerodendrum fortunatum*	3
10 mixture	10 species	3.64 ± 0.30	5.07 ± 1.44	45	2630 ± 235	*Rhodomyrtus tomentosa*	5
						*Dicranopteris dichotoma*	10
						*Clerodendrum fortunatum*	3
30 mixture	30 species	3.98 ± 0.06	3.96 ± 0.31	57	1896 ± 275	*Rhodomyrtus tomentosa*	5
						*Dicranopteris dichotoma*	12
						*Clerodendrum fortunatum*	4
Shrubland	*Rhodomyrtus tomentosa* and Baeckea frutescens L.	<2.00	–	–	–	*Rhodomyrtus tomentosa*	30
						*Dicranopteris dichotoma*	60
						*Baeckea frutescens L.*	5

**Table 2:** Soil chemical properties (0–10 cm layer) after 8 years of initial planting at Heshan Station in Jul., 2013.

Variables	EU	AC	CH	10-mixed	30-mixed	SL
Soil pH	4.28^ab^ ± 0.04	4.16^b^ ± 0.06	4.27^ab^ ± 0.03	4.28^ab^ ± 0.02	4.27^ab^ ± 0.04	4.31^a^ ± 0.04
SOM (g kg^−1^)	38.10^ab^ ± 2.67	39.20^a^ ± 4.60	33.20^ab^ ± 2.71	36.40^ab^ ± 2.76	30.00^b^ ± 1.37	39.20^a^ ± 2.91
Total N (g kg^−1^)	1.27^b^ ± 0.16	1.80^a^ ± 0.13	1.71^a^ ± 0.17	1.86^a^ ± 0.14	1.71^a^ ± 0.09	1.61^ab^ ± 0.13
Total P (g kg^−1^)	0.24^ab^ ± 0.03	0.24^ab^ ± 0.02	0.22^ab^ ± 0.02	0.23^ab^ ± 0.01	0.18^b^ ± 0.02	0.25^a^ ± 0.02
Available P(mg kg^−1^)	3.30^b^ ± 0.20	3.36^ab^ ± 0.10	3.55^ab^ ± 0.25	4.07^a^ ± 0.42	3.70^ab^ ± 0.21	3.61^ab^ ± 0.29
C/N ratio	30.79^a^ ± 0.72	21.17^bc^ ± 1.53	19.44^bc^ ± 0.59	19.55^bc^ ± 0.66	17.54^c^ ± 0.64	24.40^b^ ± 1.40

EU, *Ecucalyptus urophylla* monoculture; AC, *Acacia crassicarpa* monoculture; CH, *Castanopsis hystrix* monoculture; 10-mixed, 10 native species mixture; 30-mixed, 30 native species mixture; SL, unplanted shrubland. Different lowercase letters denote significant differences in six plantations, n = 9, *p* < 0.05.

**Table 3:** Estimated annual net N mineralization and nitrification in six restoration plantations at Heshan Station in 2013.

Parameters	EU	AC	CH	10-mixed	30-mixed	SL
N_min_	26.35^bc^ ± 3.46	52.05^a^ ± 3.40	13.19^c^ ± 3.31	48.11^a^ ± 5.33	43.40^ab^ ± 2.34	33.91^abc^ ± 3.51
N_nit_	11.00^ab^ ± 3.32	19.98^a^ ± 3.16	9.42^ab^ ± 3.04	10.52^ab^ ± 3.01	5.74^b^ ± 3.02	4.04^b^ ± 2.66
N_nit_/N_min_	41.75 ± 14.58%	38.37 ± 16.21%	71.42 ± 18.65%	21.87 ± 5.07%	13.23 ± 4.01%	11.91 ± 2.36%

AC, *Acacia crassicarpa* monoculture; EU, *Ecucalyptus urophylla* monoculture; CH, *Castanopsis hystrix* monoculture; 10-mixed, 10 native species mixture; 30-mixed, 30 native species mixture; SL, unplanted shrub land. The data were (Mean ± SD) kg N ha^−1^ year^−1^. Different lowercase letters denote significant differences in six plantations, n = 9, *p* < 0.05. N_nit_ indicated annual net nitrification and N_min_ indicated annual net N mineralization.

**Table 4:** Comparative analysis of annual N mineralization and nitrification of different aged plantations (soil 0–10 cm layer) in adjacent areas of subtropical China.

Patterns		Species name	Species Characteristics	Stand age	Net N mineralization (kg N ha^−1^ a^−1^)	Net nitrification (kg N ha^−1^ a^−1^)	Cited
Monoculture	Legumes	*Acacia crassicarpa*	Exotic, fast growth	2	25.40	19.20	Wang *et al.* (2010)^23^
		*Acacia crassicarpa*	Exotic, fast growth	8	52.05	19.98	**The present study**
		*Acacia auriculiformis*	Exotic, fast growth	13	18.36	104.40	Li *et al.* (2001)^30^*
		*Acacia auriculiformis*	Exotic, fast growth	23	70.98	112.56	Wang *et al.* (2010)^24^
		*Acacia mangium*	Exotic, fast growth	13	–	68.28	Li *et al.* (2001)^30^*
		*Acacia mangium*	Exotic, fast growth	23	57.24	89.28	Wang *et al.* (2010)^24^
	Non-legumes	*Eucalyptus urophylla*	Exotic, fast growth	2	13.50	9.98	Wang *et al.* (2010)^23^
		*Eucalyptus urophylla*	Exotic, fast growth	8	26.35	11.00	**The present study**
		*Eucalyptus citriodora*	Exotic, fast growth	23	36.60	73.86	Wang *et al.* (2010)^24^
		*Castanopsis hystrix*	Native	2	20.00	18.00	Wang *et al.* (2010)^23^
		*Castanopsis hystrix*	Native	8	13.19	9.42	**The present study**
		*Schima superba*	Native	13	104.40	10.32	Li *et al.* (2001)^30^*
		*Schima superba*	Native	23	55.74	94.14	Wang *et al.* (2010)^24^
		*Pinus elliotii*	Native	13	25.08	-	Li *et al.* (2001)^30^*
Mixture		10 trees mixture	Native	2	45.3	38.0	Wang *et al.* (2010)^23^
		10 trees mixture	Native	8	48.11	10.52	**The present study**
		30 trees mixture	Native	2	30.10	24.40	Wang *et al.* (2010)^23^
		30 trees mixture	Native	8	43.40	5.74	**The present study**
		Native species mixture	Native	23	39.78	72.90	Wang *et al.* (2010)^24^

The listed data in the above table was cited by two field-based experiments within 0–10 cm layer (Wang *et al.* 2010^23^; Wang *et al.* 2010^24^) and one laboratory-based experiment within 0–5 cm layer (Li *et al.* 2001)^30^*. The listed annual net N mineralization and nitrification in above table were calculated roughly and simply by the initial data in the published papers.
